# Danggui-Shaoyao-San (DSS) ameliorates the progression of osteoarthritis via suppressing the NF-κB signaling pathway: an *in vitro* and *in vivo* study combined with bioinformatics analysis

**DOI:** 10.18632/aging.205410

**Published:** 2024-01-08

**Authors:** Shuai Chen, Pan Kang, Zhuanglin Zhao, Hongyi Zhang, Jianliang Li, Kun Xu, Dawei Gong, Feng Jiao, Haibin Wang, Meng Zhang

**Affiliations:** 1 Guangzhou University of Chinese Medicine, Guangzhou 510405, Guangdong, China; 2 Guangzhou Hospital of Integrated Traditional and Western Medicine, Guangzhou 510800, Guangdong, China; 3 Lingnan Medical Research Center of Guangzhou University of Chinese Medicine, Guangzhou 510405, Guangdong, China; 4 Shi’s Center of Orthopedics and Traumatology, Shuguang Hospital Affiliated to Shanghai University of Traditional Chinese Medicine, Shanghai 200120, China; 5 Department of Orthopedics, The First Affiliated Hospital of Guangzhou University of Chinese Medicine, Guangzhou 510405, Guangdong, China; 6 Henan Provincial People’s Hospital, Zhengzhou University People’s Hospital, Zhengzhou 450003, Henan, China

**Keywords:** Danggui-Shaoyao-San, inflammation, bioinformatics, osteoarthritis, NF-κB

## Abstract

Background: Osteoarthritis (OA) is a common chronic age-related joint disease characterized primarily by inflammation of synovial membrane and degeneration of articular cartilage. Accumulating evidence has demonstrated that Danggui-Shaoyao-San (DSS) exerts significant anti-inflammatory effects, suggesting that it may play an important role in the treatment of knee osteoarthritis (KOA).

Methods: In the present study, DSS was prepared and analyzed by high-performance liquid chromatography (HPLC). Bioinformatics analyses were carried out to uncover the functions and possible molecular mechanisms by which DSS against KOA. Furthermore, the protective effects of DSS on lipopolysaccharide (LPS)-induced rat chondrocytes and cartilage degeneration in a rat OA model were investigated *in vivo* and *in vitro*.

Results: In total, 114 targets of DSS were identified, of which 60 candidate targets were related to KOA. The target enrichment analysis suggested that the NF-κB signaling pathway may be an effective mechanism of DSS. *In vitro*, we found that DSS significantly inhibited LPS-induced upregulation of inducible nitric oxide synthase (iNOS), cyclooxygenase-2 (COX-2), interleukin-6 (IL-6), matrix metalloproteinase-3 (MMP3), and matrix metalloproteinase-13 (MMP13). Meanwhile, the degradation of collagen II was also reversed by DSS. Mechanistically, DSS dramatically suppressed LPS-induced activation of the nuclear factor kappa B (NF-κB) signaling pathway. *In vivo*, DSS treatment prevented cartilage degeneration in a rat OA model.

Conclusions: DSS could ameliorate the progression of OA through suppressing the NF-κB signaling pathway. Our findings indicate that DSS may be a promising therapeutic approach for the treatment of KOA.

## INTRODUCTION

Osteoarthritis (OA) is one of the most common age-related degenerative joint diseases, characterized by progressive cartilage destruction, subchondral remodeling, and synovial inflammation [1]. The main clinical symptoms and signs of knee osteoarthritis include joint pain, stiffness, muscle weakness, and even deformity with reduced joint motion. Due to the aging of the global population, the number of individuals suffering from OA is expected to significantly increase in the coming decades [2]. OA leads to considerable disability and a reduction in quality of life, and places huge socioeconomic costs [3, 4]. At present, treatment options include oral medication, intra-articular injection, and surgery intended to relieve symptoms or prevent functional decline [5, 6]. Although some patients benefit from treatment, joint damage can still progress even after clinical remission. Therefore, a better understanding of the mechanisms of OA will provide new therapeutic strategies to prevent the onset or slow the progression of OA.

Danggui-Shaoyao-San (DSS) is a well-known herbal formula of traditional Chinese medicine, which consists of Angelica sinensis (Oliv.) Diels. (Dang-gui), Paeonia lactiflora Pall. (Bai-shao), Atractylodes macrocephala Koidz (Bai-zhu), Poria cocos (Schw.) Wolf (Fu-ling), Alisma orientalis (Sam.) Juzep (Ze-xie) and Ligusticum chuanxiong Hort. (Chuan-xiong). Mounting evidence demonstrates that DSS possesses multiple beneficial properties, including anti-inflammatory, antioxidant, and neuroprotective effects, making it a promising therapy option for various diseases [7–11]. Recently, numerous studies highlight the importance of DSS in suppressing inflammation. It has been reported that DSS significantly attenuates inflammation in metabolic syndrome rats via downregulating the expression of NLRP12, TNF-α, and IL-33 [12]. Previous study indicated that DSS can prevent brain injury and inflammation via suppressing the secretion of TNF-α, IL-1β and IL-8, as well as activation of the NF-κB signaling pathway [13]. Moreover, DSS treatment could reduce the release of various inflammatory cytokines in HUVECs via the reciprocal MAPK/NF-κB signaling pathways, which makes it an attractive therapeutic approach for preventing atherosclerotic cardiovascular diseases [14]. However, little is known regarding the potential role of DSS in the treatment of OA.

In the present study, we screened the potential targets and pathways associated with DSS for treating OA by using bioinformatics tools. We further analyzed the underlying pharmacological mechanisms of DSS against OA and validated our findings by conducting *in vitro* and *in vivo* experiments.

## MATERIALS AND METHODS

### Screening potential targets of DSS

The ingredients of six drugs in DSS were extracted from the Traditional Chinese Medicine Systems Pharmacology Database and Analysis Platform (TCMSP, https://old.tcmsp-e.com/tcmsp.php) [15]. In our present study, the bioactive ingredients were filtered using ADME (absorption, distribution, metabolism, and excretion) evaluation systems. For further research, only those ingredients fulfilling the established criterion of both oral bioavailability (OB ≥ 30%) and drug-likeness (DL ≥ 0.18) were considered as bioactive ingredients. The targets information linked with the bioactive ingredients was collected from the TCMSP database. Bioactive ingredients were excluded if there was no target information available.

### Collection of KOA-related targets

The targets associated with knee osteoarthritis (KOA) were gathered from the GeneCards database (https://www.genecards.org/) [16] and the DisGeNet database (http://www.disgenet.org/) [17]. After removing the duplicates, those targets related to KOA were finally obtained.

### Screening of candidate targets and construction of the protein–protein interaction network

We uploaded DSS-related targets and KOA-related targets to TBtools, and the common genes shared by them were identified as candidate targets, The Venn diagram was generated using Venn in TBtools. After that, candidate targets were submitted to STRING database (https://string-db.org/) to construct protein–protein interaction (PPI) network with the default settings. Furthermore, the CytoHubba plugin in Cytoscape was used to identify the hub genes in the PPI network.

### Functional enrichment analysis

Candidate targets were submitted to the DAVID database (https://david.ncifcrf.gov/) [18] to perform Gene Ontology (GO) and Kyoto Encyclopedia of Genes and Genomes (KEGG) pathway enrichment analysis. The GO enrichment analysis consists of molecular function (MF), biological process (BP), and cellular component (CC). The results of the enrichment analysis were presented with a bar chart using an online webtool.

### The preparation of the DSS

The drug composition of DSS refers to the original formula: Dang-gui (3g), Bai-shao (16g), Bai-zhu (4g), Fu-ling (4g), Ze-xie (8g) and Chuan-xiong (8g). All drugs were purchased from KangMei Pharmaceutical Co., Ltd (Guangdong, China). The preparation of the water extract of DSS was as follows: 200 g drugs were mixed and soaked in 1 L double-distilled water for 2 h, boiled at high heat for 30 min and simmered for another 1 h, the two obtained filtrates were combined and concentrated to 1.7 g/mL by a rotary evaporator. All liquids were centrifuged (8000x g, 10 min) and filtered with a 0.22 μm filter for liquid chromatography analysis and subsequent experiments.

### High-performance liquid chromatography (HPLC) analysis

High-performance liquid chromatography (HPLC) analysis was performed using a Waters Synapt G2-Si quadrupole time-of-flight mass. The chromatographic separations of DSS were performed on an Acquity UPLC HSS T3 column at 35° C, with a mobile-phase flow rate of 0.25 mL/min. The injection volume was 20 μL. The mobile phase consisted of deionized water containing 0.1% formic acid (A) and acetonitrile containing 0.1% formic acid (B) with the following gradient elution: 0–15 min, 100% A; 15–50 min, 80% A and 20% B; 50–70 min, 100% B; 70 min, 100% A.

### Chondrocyte isolation, culture, and treatment

As described previously, chondrocytes were isolated from 4-week-old Sprague Dawley rats and maintained in DMEM/F-12 medium supplemented with 10% fetal bovine serum (FBS) and a 1% antibiotic mixture (penicillin and streptomycin) [19]. Briefly, cartilage was cut into small pieces and then digested with 0.25% trypsin at 37° C for 30 min. Cartilage pieces were digested with 2mg/mL collagenase type II for 4 h, followed by centrifugation at 1000 rpm for 5 min. After discarding the supernatant, the pellets were resuspended in DMEM/F-12. Cells in passage 2 were used for subsequent experiments. Chondrocytes were pretreated with different concentrations of DSS for 24 h, followed by stimulation with or without LPS (10 μg/mL) for 24 h according to the study design.

### Cell viability

The cell viability was measured using the Cell Counting Kit-8 (Bimake, USA). Briefly, chondrocytes were seeded into 96-well plates and treated with different concentrations of DSS (0.5, 1, 1.5, and 2 mg/mL). After 24 h or 48 h of incubation, 10 μl of CCK-8 solution was added into each well and incubated for another 1 h. The absorbance of each well was examined at 450 nm with a microplate reader.

### Quantitative real-time polymerase chain reaction (qRT-PCR)

Total RNA was isolated from cells with a NucleoZOL reagent (Machery-Nagel GmbH, Germany) following the manufacturer’s introduction, and cDNA was synthesized with a reverse transcriptase kit. qRT-PCR was carried out using Hieff® qPCR SYBR Green Master Mix (YEASON, China) on an ABI Stepone plus real-time PCR system (Applied Biosystems, CA). GAPDH was used as a normalization control to standardize the expression of each target gene. The primer sequences used in this study were listed in Supplementary Table 1.

### Western blot analysis

Cells were washed with ice-cold PBS and lysed in RIPA lysis buffer supplemented with protease and phosphatase inhibitors. The protein concentrations were quantified using a BCA protein assay kit (Beyotime, China). Equal amounts of protein from each sample were loaded and separated on SDS-PAGE gels, then transferred onto PVDF membranes. The membranes were subsequently blocked in 5% BSA for 1 h at room temperature and incubated with primary antibodies overnight at 4° C, followed by corresponding secondary antibody (goat anti-mouse, E030110-01, EarthOx; goat anti-rabbit, E030120-01, EarthOx) and incubation at room temperature for 1 h. The protein bands were detected and visualized using a Bio-Rad gel imaging system. The primary antibodies used were as follows: anti-iNOS (18985-1-AP, Proteintech), anti-COX2 (12375-1-AP, Proteintech), anti-MMP3 (17873-1-AP, Proteintech), anti-MMP13 (18165-1-AP, Proteintech), anti-p-IκBα (2859, CST), anti-IκBα (4814, CST), anti-p-p65 (3033, CST), anti-p65 (8242, CST), and anti-GAPDH (17873-1-AP, Proteintech).

### Immunofluorescence assay

For detecting collagen II, chondrocytes were pretreated with DSS for 24 h and then treated with or without LPS for another 24 h. For detecting p65, the stimulation time of LPS was only 2 h. At indicated time points, cells were harvested, washed, fixed, permeabilized, and blocked following the manufacturer’s protocol. Then the cells were incubated with collagen II (CY1019, Abways) and p65 (8242, CST) overnight at 4° C respectively, followed by incubation with corresponding secondary antibodies. Finally, the cells were mounted with DAPI contained fluorescent mounting solution (F6057, Sigma-Aldrich). The images were observed and captured with a fluorescence microscope (Olympus).

### Animal experiments

Six-week-old SD male rats were purchased from the Animal Center of Guangzhou University of Chinese Medicine (No.44005800013231). The rats were randomly divided into four groups: sham group, OA group, DSS-L group, and DSS-H group. The rat OA model was established using a modified Hulth method based on previous reports [20]. In brief, after administration of the anesthesia, the medial collateral ligament was cut to allow access to the joint cavity of the right knee, followed by excising the medial meniscus and transected anterior cruciate ligament. Rats in the sham group underwent merely the same skin incision operation. The dosage of DSS was calculated according to the dose equivalents between humans and laboratory animals based on ratios of body surface area as described in a previous study [21]. After surgery, rats were treated with saline or DSS (1.7 g/kg for DSS-L group and 3.5 g/kg for DSS-H group) once a day by oral gavage for 6 weeks.

### Histological analysis and immunohistochemistry

The harvested samples from each group were fixed with 4% paraformaldehyde for 24 h, decalcified with 10% EDTA for 4 weeks, and hydrated through a graded ethanol series. Eventually, the paraffin-embedded specimens were sectioned into 5 μm thick sections, which were subsequently stained with hematoxylin and eosin (H&E) staining and Safranin O staining for histological analysis. Additionally, the expression of MMP13 and p65 were detected using immunohistochemical analysis according to a standard procedure.

### Statistical analysis

The experimental results were displayed as mean ± standard deviation (SD) and statistically analyzed with GraphPad Prism 8.0 (GraphPad). Comparisons among multiple groups were analyzed by one-way analyses of variance (ANOVA). The P-values less than 0.05 were considered statistically significant.

## RESULTS

### Candidate targets and PPI network analysis

**Figure 1:**
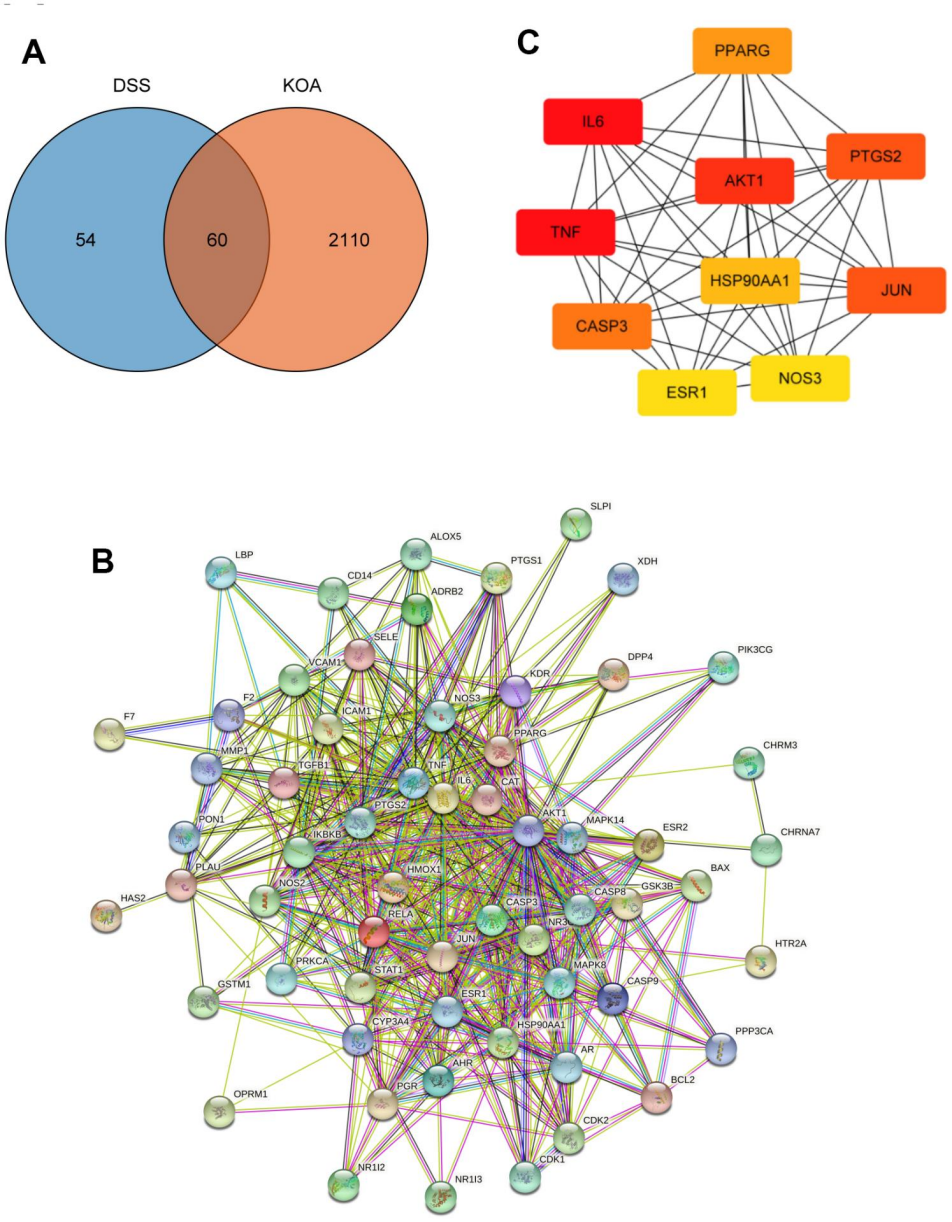
**Network pharmacology analysis.** (**A**) A Venn diagram was performed to obtain the candidate targets of the DSS and KOA. (**B**) PPI network of the candidate targets. (**C**) The top 10 hub genes ranked by degree.

In total, 51 bioactive ingredients were identified from the TCMSP database based on the screening criteria, and 114 related targets were obtained after removing duplicates. Meanwhile, a total of 2170 targets associated with KOA were obtained from GeneCards and DisGeNet databases after integration and deduplication. As shown in Venn diagram (Figure 1A), there were 60 targets that overlapped between the DSS ingredients and KOA-related targets. The PPI network was composed of 60 nodes and 550 edges, the average node degree was 18.3 (Figure 1B). By using the CytoHubba plugin, 10 genes with the highest degree were selected as hub genes that may play an important role in the treatment of KOA with DSS, including IL6, TNF, AKT1, PTGS2, JUN, CASP3, PPARG, HSP90AA1, NOS3, and ESR1 (Figure 1C).

### Functional enrichment analysis

**Figure 2:**
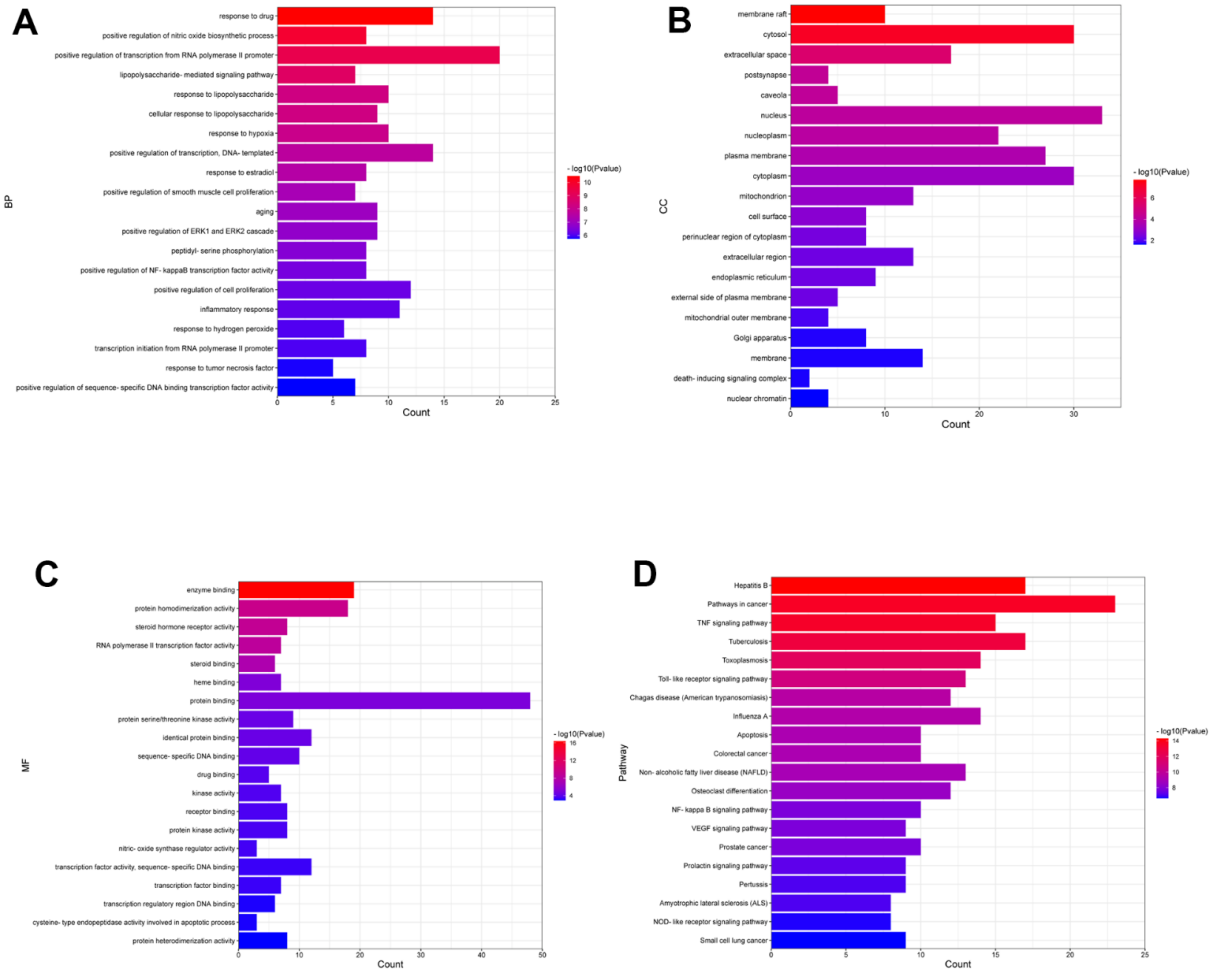
**Enrichment analysis of the candidate targets.** (**A**) Bar chart displaying the top 20 enriched terms of biological processes (BP), ranked by P-value. (**B**) Bar chart displaying the top 20 enriched terms of cell component (CC), ranked by *P*-value. (**C**) Bar chart displaying the top 20 enriched terms of molecular function (MF), ranked by *P*-value. (**D**) The top 20 enriched KEGG pathways ranked by *P*-value are presented in the bar chart.

To explore the molecular mechanism of DSS against OA, functional enrichment analysis of candidate targets was carried out by using the David database. The top 20 most significant terms classified into BP, CC, and MF were displayed as a bar chart (Figure 2A–2C). We observed that the GO terms were highly correlated with endogenous and exogenous inflammatory responses, such as positive regulation of nitric oxide biosynthetic process, lipopolysaccharide-mediated signaling pathway, response to lipopolysaccharide, cellular response to lipopolysaccharide, positive regulation of NF-kappaB transcription factor activity, inflammatory response, and response to tumor necrosis factor. The KEGG pathway enrichment analysis showed that DSS treatment KOA was primarily involved in Pathways in cancer, TNF signaling pathway, Toll-like receptor signaling pathway, Apoptosis, and NF-kappa B signaling pathway. The top 20 most significant KEGG pathways were shown in (Figure 2D).

### HPLC analysis of the components of DSS

**Figure 3:**
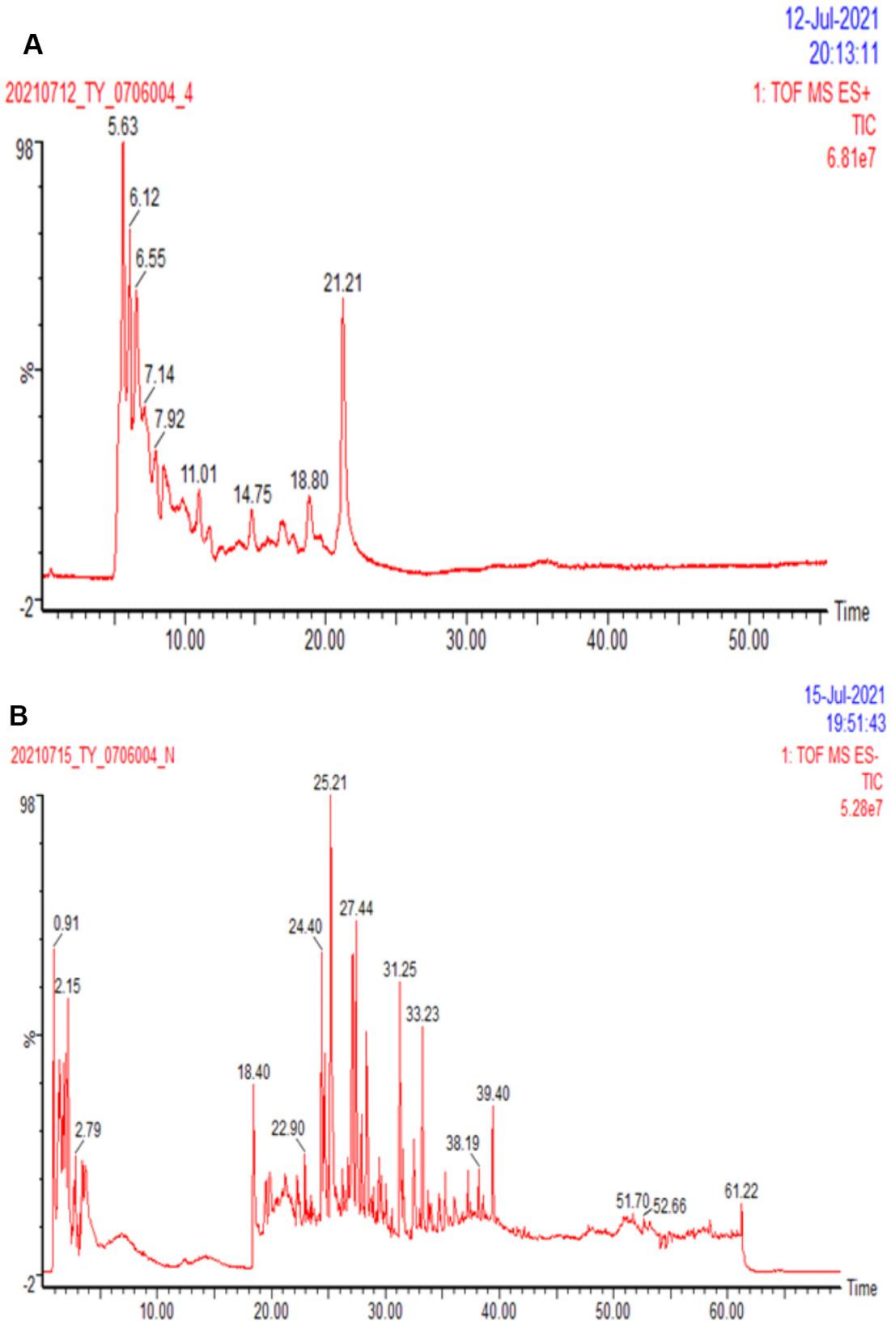
**Ion flow diagram of DSS detected by HPLC.** (**A**) The representative total ion chromatography of sample in positive ion mode. (**B**) The representative total ion chromatography of sample in negative ion mode.

HPLC was applied to analyze the chemical compositions of DSS qualitatively and quantitatively. As can be seen in Figure 3, the top ten compounds are as follows: Ginseng Trisaccharide (5.67%), Ephedra F (5.549%), Galloylpaeoniflorin (5.143%), Forsythiaside A (4.987%), β-Gentiobiose (3.994%), Arginine (3.617%), Paeonoside J (3.600%), Stachyose (2.303%), Mueculoside E (2.151%), Echinothiophene (1.989%). Other compounds information could be found in Supplementary Table 2.

### Effects of DSS on cell viability

**Figure 4:**
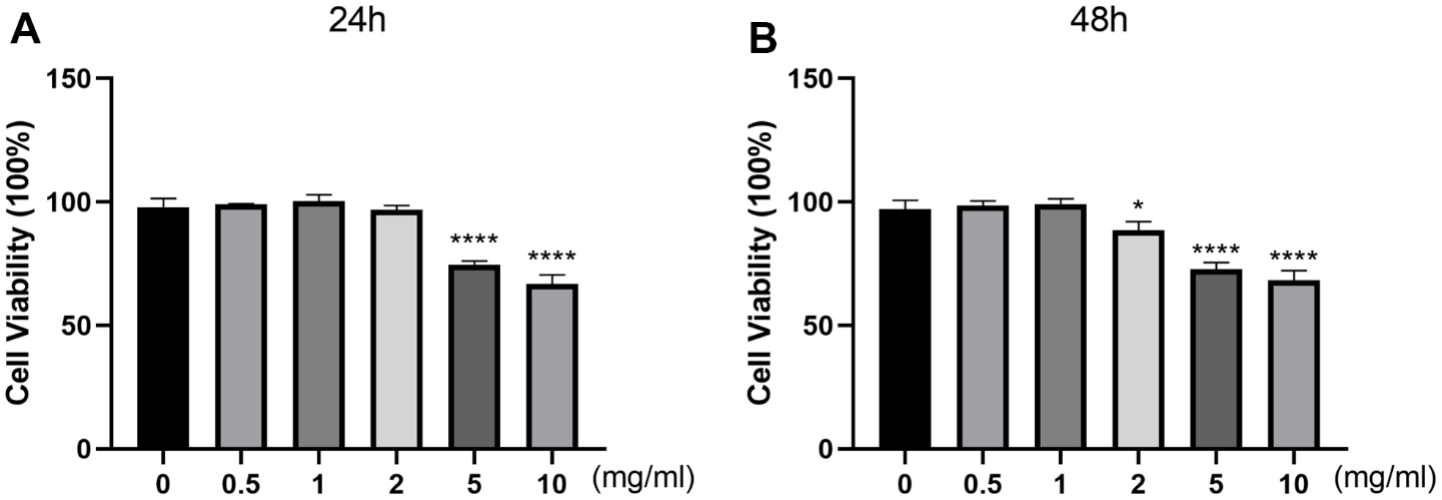
**Effects of DSS on chondrocyte viability.** The cytotoxicity of DSS on chondrocytes was examined at various concentrations for 24 h (**A**) and 48 h by CCK-8 assay (**B**), respectively. ^*^p < 0.05, ^****^p < 0.0001 vs. control group, n = 3.

To evaluate the cytotoxic effect of DSS on the viability of chondrocytes, cells were treated with different concentrations of DSS for 24 h or 48 h, followed by the CCK-8 detection. As shown in Figure 4A, DSS at the concentrations of 0.5, 1, 2 mg/mL exerted no cytotoxicity to chondrocytes, while high concentrations (5 and 10 mg/mL) of DSS reduced cell viability significantly. As shown in Figure 4B, DSS treatment at the concentrations of 0.5 and 1 mg/mL did not affect the viability of chondrocytes. Based on this, DSS at concentrations of 0.5 and 1 mg/mL were used in further experiments.

### The protective effects of DSS on LPS-induced inflammatory reaction in rat chondrocytes

**Figure 5:**
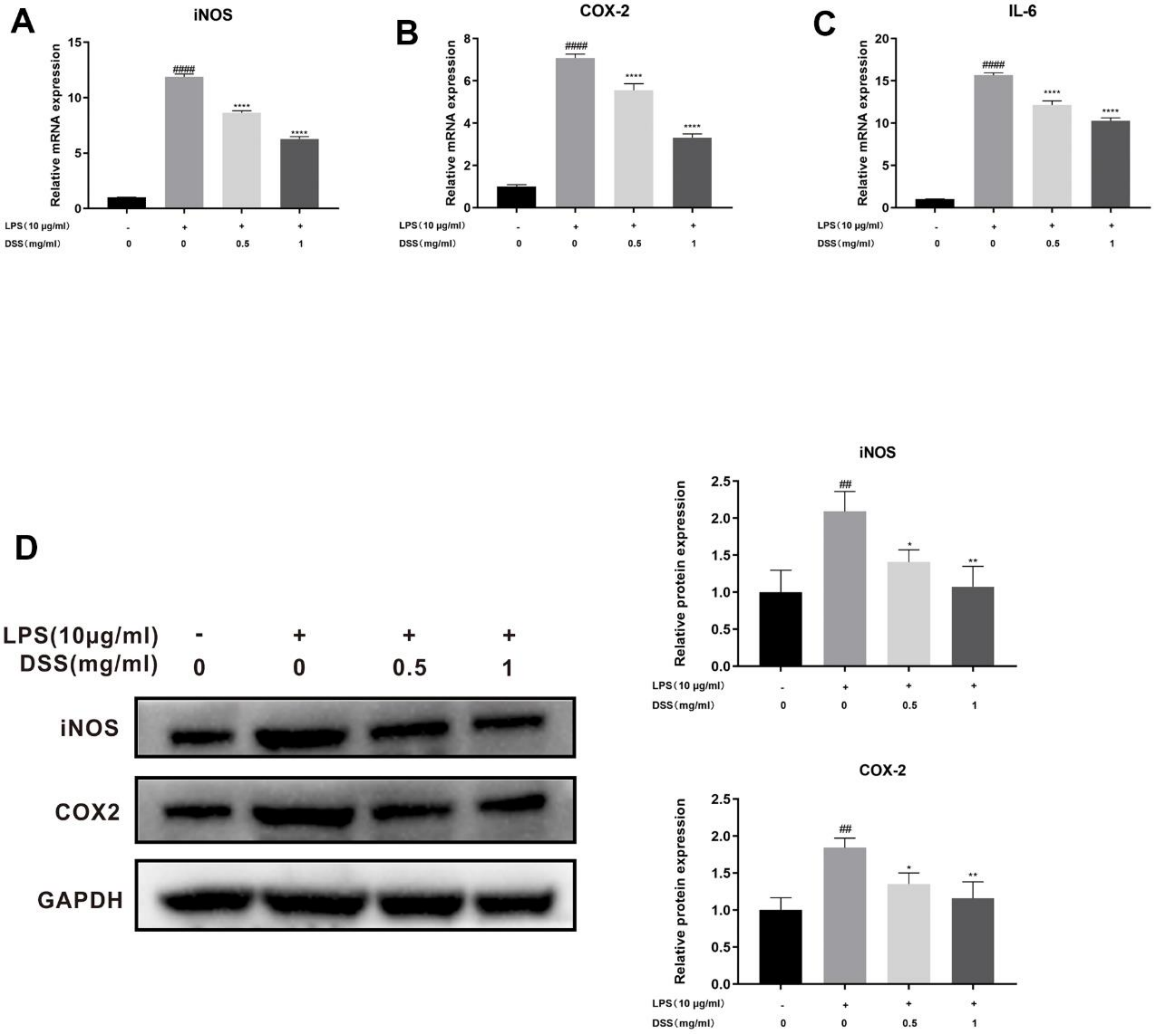
**DSS inhibited LPS-induced inflammation in chondrocytes.** The chondrocytes were pretreated with DSS for 24 h and stimulated with or without LPS for 2 h. (**A**–**C**) The mRNA expression levels of iNOS, COX-2, and IL-6 were detected by qRT-PCR. (**D**) The protein levels of iNOS and COX-2 were measured by western blot. ^##^*p* < 0.01, ^####^*p* < 0.0001 vs. control group; ^*^p < 0.05, ^**^p < 0.01, ^****^p < 0.0001 vs. LPS alone treatment group, n = 3.

Subsequently, we assessed the effect of DSS on LPS-induced chondrocyte inflammation by qRT-PCR and Western blot analysis. As shown in Figure 5A–5C, qRT-PCR results showed that the mRNA expression of iNOS, COX-2, and IL-6 were upregulated after LPS stimulation. However, DSS treatment dramatically suppressed the production of iNOS, COX-2, and IL-6 in a concentration-dependent manner. Similarly, in line with the qRT-PCR results, the western blot analysis also confirmed that DSS treatment concentration-dependently inhibited the inflammation-related protein levels of iNOS and COX-2 (Figure 5D). Taken together, these results suggest that DSS could decrease the production of inflammatory cytokines at the concentration of 0.5 and 1 mg/mL.

### DSS attenuated LPS-induced extracellular matrix (ECM) degradation in rat chondrocytes

**Figure 6:**
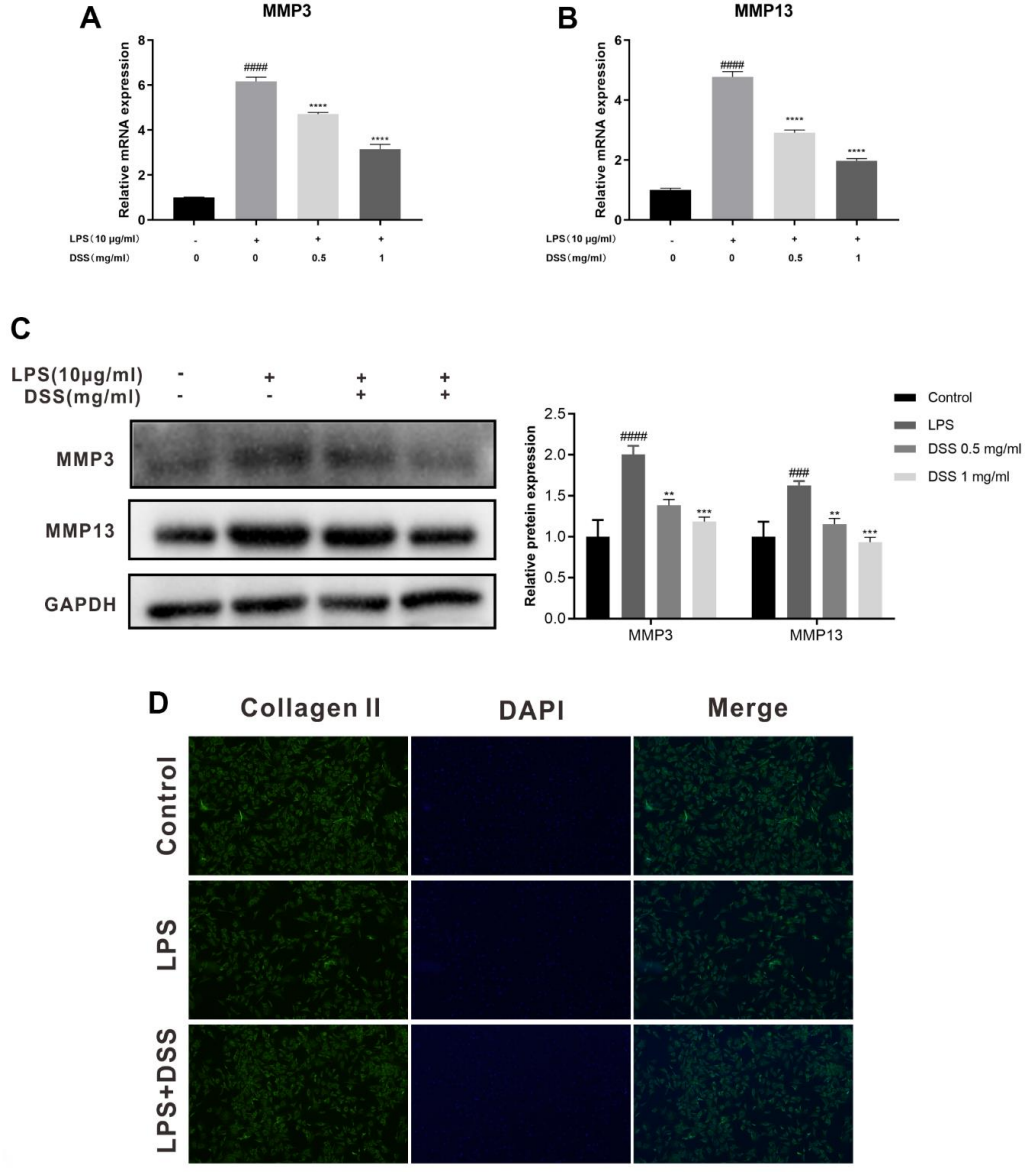
**DSS suppressed ECM degradation in rat chondrocytes.** The chondrocytes were pretreated with DSS for 24 h and stimulated with or without LPS for 24 h. (**A**, **B**) The mRNA expression levels of MMP3 and MMP13 were detected by qRT-PCR. (**C**) The protein levels of MMP3 and MMP13 in chondrocytes were measured by western blot. (**D**) Representative immunofluorescence image of collagen II, and the fluorescence intensities were quantified by Image J. ^##^*p* < 0.01, ^###^*p* < 0.001, ^####^*p* < 0.0001 vs. control group; ^*^p < 0.05, ^**^p < 0.01, ^***^p < 0.001, ^****^p < 0.0001 vs. LPS alone treatment group, n = 3.

Then, we investigated the effect of DSS on extracellular matrix (ECM) metabolic equilibrium induced by LPS in rat OA chondrocytes. As presented in Figure 6A–6C, LPS markedly upregulated the expression of MMP3 and MMP13 compared with the control group. Whereas, DSS inhibited the overexpression of MMP-3 and MMP13 induced by LPS in a concentration-dependent manner respectively. Additionally, the results of immunofluorescence staining showed that DSS manifestly inhibited the protein degradation of collagen-II and protected against the adverse effects caused by LPS (Figure 6D). Altogether, these findings demonstrated that DSS protects rat chondrocytes from LPS-mediated ECM degradation.

### DSS suppressed LPS-induced NF-κB activation in rat chondrocytes

**Figure 7:**
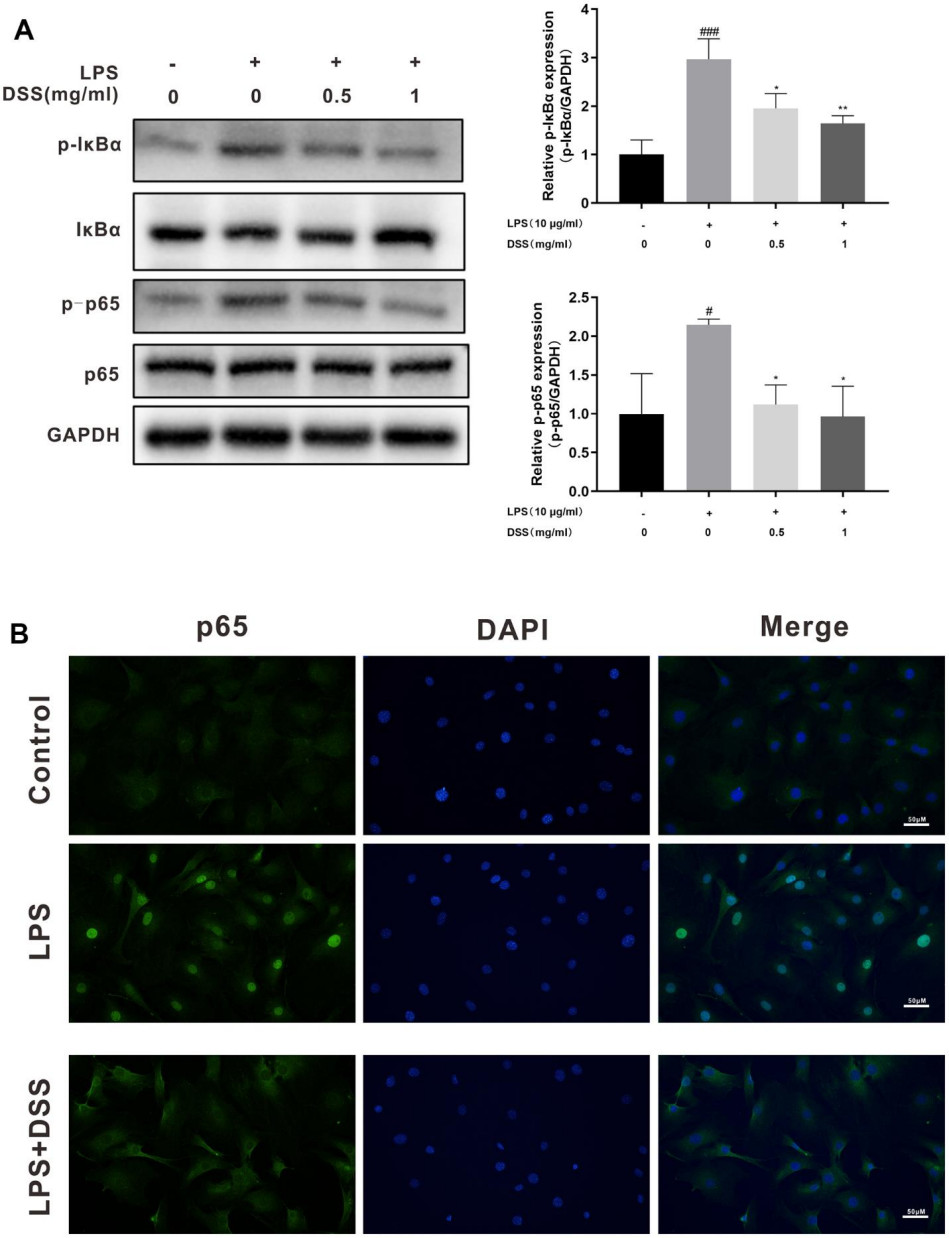
**DSS inhibited LPS mediated activation of the NF-κB pathway.** The chondrocytes were pretreated with DSS for 24 h and stimulated with or without LPS for 2 h. (**A**) The protein levels of p-IκBα, IκBα, p-p65 and p65 were assessed by western blot. (**B**) The nuclei translocation of p65 was analyzed by the immunofluorescence staining. ^#^*p* < 0.05, ^###^*p* < 0.001 vs. control group; ^*^p < 0.05, ^**^p < 0.01 vs. LPS alone treatment group, n = 3.

To further explore the potential mechanism of the anti-inflammatory action of DSS, we performed western blot analysis and immunofluorescence to detect changes in NF-κB signaling activity. As shown in Figure 7A, LPS dramatically increased the phosphorylation of NF-κB p65 and IκBα in chondrocytes. Nevertheless, pretreatment with various concentrations of DSS (0.5 and 1 mg/mL) effectively reduced LPS-induced phosphorylation of NF-κB p65. Moreover, when chondrocytes were stimulated with LPS, remarkable degradation of IκBα occurred. By contrast, pretreatment with DSS reversed this effect. In addition, results of immunofluorescence staining further verified the suppressive effect of DSS on LPS-induced nuclear translocation of NF-κB p65 (Figure 7B).

### DSS ameliorates OA progression in a rat OA model

**Figure 8:**
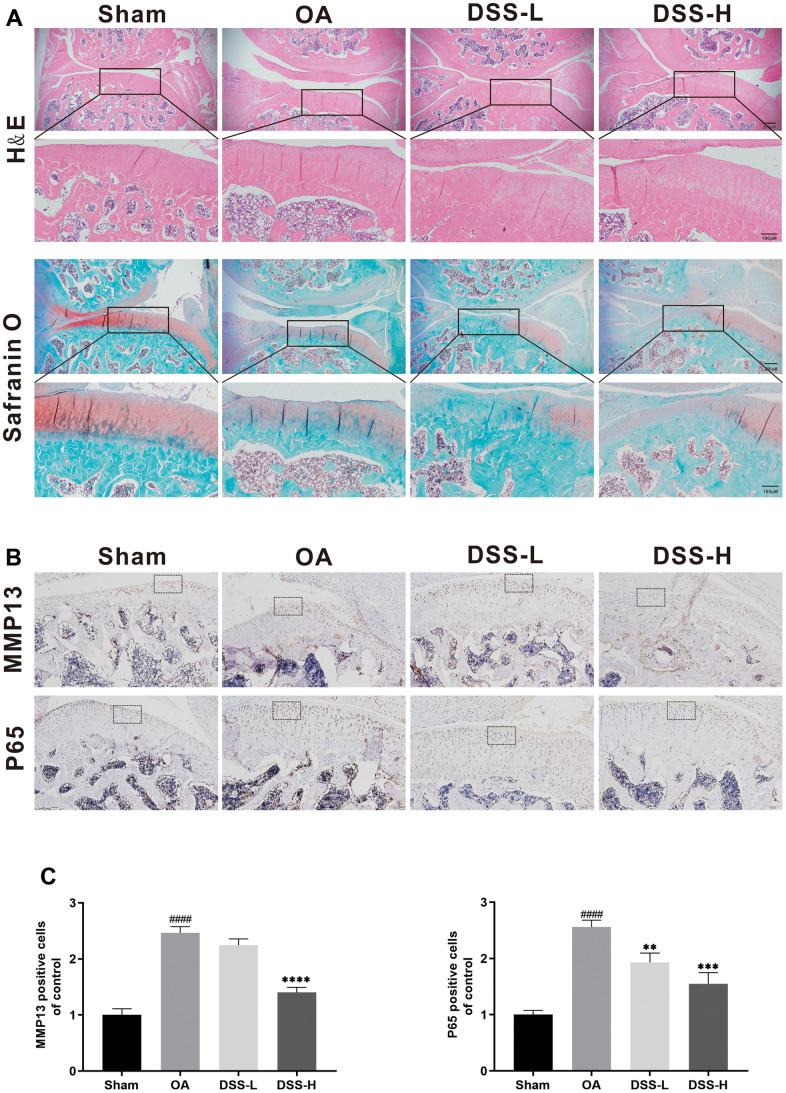
**DSS ameliorated OA progression in a rat OA model *in vivo*.** (**A**) Representative H&E and Safranin O staining of cartilage and synovitis from different experimental groups. (**B**) Immunohistochemical staining of MMP13 and P65 was employed to evaluate the effect of DSS on the cartilage. (**C**) Quantitative analysis of positive cells in cartilage. ^####^*p* < 0.0001 vs. sham group; ^**^p < 0.01, ^***^p < 0.001, ^****^p < 0.0001 vs. OA group, n = 6.

To further explore the effect of DSS on the progress of OA *in vivo*, a surgically induced rat OA model was established, followed by gavage of DSS or saline for 6 weeks. Hematological examination of the cartilage was performed by using H&E and Safranin O staining. The OA group exhibited significant pathologic changes, including severe cartilage destruction, cartilage erosion, and apparent chondrocyte hypocellularity when compared with the sham group (Figure 8A). In contrast, treatment with DSS remarkably reduced cartilage destruction compared with the OA group, especially in the DSS-H group. Additionally, immunohistochemical results showed that the number of MMP13 and p65 positive cells was increased in the OA group, while the protein expression of MMP13 and p65 reduced significantly in the DSS-H group (Figure 8B, 8C). Altogether, these data demonstrated that DSS efficiently suppressed OA progression *in vivo*.

## DISCUSSION

OA is the most common disabling joint disease associated with inflammation and seriously impacts the daily life of the elderly [22]. Currently, effective treatment approaches against OA are still limited. It is urgent to find new drugs and targets that can significantly delay the progression of OA. DSS was first recorded in Synopsis of Golden Chamber, and its beneficial effects have now been widely demonstrated in various diseases [14, 23]. It has been demonstrated in previous studies that several botanical drugs or their bioactive components in DSS have anti-inflammatory activity and thus can be used in the treatment of OA [24–26]. In the present study, bioinformatics analysis and experimental validation were performed to investigate the potential role of DSS in inhibiting LPS-induced inflammatory injury in chondrocytes and to explore the molecular mechanisms responsible for this regulation.

Through network pharmacology analysis, active ingredients in DSS and related targets were identified, and 60 intersection targets of DSS and KOA were obtained. Subsequently, the top 10 hub genes for the treatment of KOA with DSS were identified, including IL6, TNF, AKT1, PTGS2, and CASP3. IL-6 and TNF are important pro-inflammatory cytokines participating in the mediation of inflammation and immune response. It has been reported that the level of IL-6 was significantly increased in the synovial fluid of patients with OA [27]. Increased expression of TNF in chondrocytes results in the activation of NF-κB pathway, which in turn promotes the expression of inflammatory mediators and matrix-degrading enzymes [28]. PTGS2, which is also known as COX-2, serves as a critical enzyme for the synthesis of prostaglandins and plays a key role in the development of OA [29]. To determine the potential mechanisms of DSS for treating OA, we conducted functional enrichment analysis on the 60 candidate targets involved in both KOA and DSS. The GO enrichment analysis showed that these candidate targets could modulate a variety of biological processes, but were mainly involved in the biological processes associated with inflammatory responses. Among the most significantly enriched KEGG pathways, TNF signaling pathway, Toll-like receptor signaling pathway, Apoptosis, and NF-kappa B signaling pathway are highly related to the onset and progression of OA [30–32]. Growing evidence has demonstrated the important role of the TNF signaling pathway in cell growth, differentiation, apoptosis, and inflammation. Dysregulation of the TNF signaling pathway has been implicated in the pathogenesis of a variety of diseases, including rheumatoid arthritis and inflammatory bowel disease [33]. In addition, persistent activation of the NF-κB pathway can accelerate inflammation-related cartilage damage. The bioinformatics analysis revealed that DSS exerts anti-KOA activity with features of multi-component, multi-target, and multi-pathway, which provides a theoretical basis for the treatment of KOA. Meanwhile, we found that DSS may act mainly on inflammation-related targets and pathways to treat KOA. Given the importance of inflammation in the pathogenesis of OA, we speculate that the mechanism of DSS for the treatment of KOA may be related to the inhibition of inflammation-related signaling pathways, thereby reducing the inflammatory response.

In order to further validate the bioinformatics predictions, we investigated the protective effect of DSS on chondrocytes *in vitro* by establishing the LPS-induced chondrocyte injury model. LPS was regarded as the inflammatory stimulant that mimicked the inflammatory environment in an OA joint [34]. Substantial evidence has indicated that LPS stimulation leads to an imbalance between catabolism and anabolism in chondrocytes by increasing the expression of COX-2 and iNOS, as well as promoting the production of ECM-degrading proteinases, such as MMP3, MMP13, and ADAMTS [35]. MMP3 and MMP13 have long been demonstrated to be highly expressed in the cartilage of patients who are suffering from OA, which results in the progression of OA [36]. In line with the results of previous studies, we found that LPS stimulation significantly upregulated the expression of iNOS and COX-2 both in mRNA and protein levels [37]. Moreover, the stimulation of LPS also resulted in a significant increase of MMP3 and MMP13, as well as a decrease of collagen II. In contrast, DSS treatment dramatically ameliorated the negative effects induced by LPS in chondrocytes. Collectively, these findings revealed that DSS could effectively inhibit inflammation, degradation of ECM, and promote ECM synthesis.

Previous studies have shown NF-κB is involved in the regulation of various biological processes and plays a vital role in inflammatory response [38]. Various stimulus, including several inflammatory mediators and LPS, can activate the NF-κB signaling pathway. The abnormal activation of the NF-κB signaling pathway is closely correlated with the onset, development, and progression of OA [39]. When chondrocytes are stimulated by LPS, the NF-KB signaling pathway is activated, resulting in the production of inflammatory mediators and matrix catabolic enzymes, which promote chondrocyte apoptosis and ECM degradation [40]. Therefore, targeting strategies that block the NF-κB signaling pathway may provide promising approaches for OA therapy [41, 42]. In the present study, we investigated the effect of DSS on the NF-κB signaling pathway. Our results demonstrated that DSS treatment significantly reduced the phosphorylation of IKBα and P65, suggesting that DSS effectively inhibited LPS-induced activation of the NF-κB signaling pathway. Furthermore, DSS suppressed nuclear translocation of p65 as observed with immunofluorescence staining. Interestingly, our results are in accordance with a previous study which demonstrated that DSS inhibited the NF-κB pathway in LPS-induced HUVEC injury model [14]. Hence, the mechanism by which DSS exerts its therapeutic effect may be accounted for by this inhibition of chondrocyte inflammation.

Following the definite demonstration of DSS’s protective effect *in vitro*, we further evaluated the effect of DSS on cartilage degeneration by using a rat OA model *in vivo*. In this experiment, the OA group showed severe cartilage surface destruction, which was partially reversed by treatment with DSS, particularly in the high-dose group. In addition, DSS significantly reduced the protein expression of MMP13 and p65 in OA rat articular cartilages, suggesting that DSS can effectively modulate NF-κB mediated inflammatory responses to attenuate the progression of OA. However, there were several limitations that had to be taken into account in this study. Firstly, further studies will be necessary to determine whether DSS affects other targets that are capable of delaying the progression of OA or whether other regulatory mechanisms are also involved. Secondly, clinical studies are needed to determine the appropriate concentration and duration of treatment with DSS for OA, as well as to assess its safety.

In conclusion, our findings revealed that DSS significantly reduced inflammatory response induced by LPS *in vitro* and effectively attenuated OA-induced cartilage degeneration in a rat model via inhibiting the NF-κB pathway. Collectively, in spite of the need for further study, the data obtained in this study confirm the potential benefits of DSS in OA and provide a promising treatment strategy.

## Supplementary Material

Supplementary Table 1

Supplementary Table 2
