# Supplementary material for: Danggui-Shaoyao-San (DSS) ameliorates the progression of osteoarthritis via suppressing the NF-κB signaling pathway: an in vitro and in vivo study combined with bioinformatics analysis
Source: Aging (Albany NY). 2024 Jan 8;16(1):648–64. doi: 10.18632/aging.205410 (PMC10817397; doi:10.18632/aging.205410)
Supplement: Supplementary Table 1 [file aging-16-205410-s001.pdf]

SUPPLEMENTARY TABLES

Supplementary Table 1. Primer sequences used for qRT-PCR analysis.

Gene name	Forward primer	Reverse primer
Rat-iNOS	CCTTACGAGGCGAAGAAGGACAG	CAGTTTGAGAGAGGAGGCTCCG
Rat-COX-2	GATGACGAGCGACTGTTCCA	CAATGTTGAAGGTGTCCGGC
Rat-IL-6	AAGCCAGAGTCATTCAGAGC	GTCCTTAGCCACTCCTTCTG
Rat-MMP3	TCTTCCTCTGAAACTTGGCG	AGTGCTTCTGAATGTCCTTCG
Rat-MMP13	AGCTCCAAAGGCTACAACTTAT	GTCTTCATCTCCTGGACCATAG
Rat-GAPDH	TGGAGTCTACTGGCGTCTT	TGTCATATTTCTCGTGGTTCA
